# Antibodies to malaria vaccine candidates are associated with chloroquine or sulphadoxine/pyrimethamine treatment efficacy in children in an endemic area of Burkina Faso

**DOI:** 10.1186/1475-2875-11-79

**Published:** 2012-03-22

**Authors:** Amidou Diarra, Issa Nebie, Alfred Tiono, Issiaka Soulama, Alphonse Ouedraogo, Amadou Konate, Michael Theisen, Daniel Dodoo, Alfred Traore, Sodiomon B Sirima

**Affiliations:** 1 Centre National de Recherche et de Formation sur le paludisme, o1 BP 2208 Ouagadougou 01, Burkina Faso; 2 Department of Clinical Biochemistry, Statens Serum, 5, Orestads Boulevard, DK-2300 Copenhagen, Denmark; 3 Noguchi Memorial Institute for Medical Research, Legon, P.O. Box M44, Accra, Ghana; 4 UFR Sciences de la Vie et de la Terre (SVT), Université de Ouagadougou, Ouagadougou, Burkina Faso; 5 Groupe de Recherche Action en Santé, 06 BP 10248 Ouagadougou 06, Burkina Faso

**Keywords:** Antibodies, Chloroquine, Sulphadoxine/pyrimethamine, MSP3, GLURP, MSP1-19

## Abstract

**Background:**

Patient immune status is thought to affect the efficacy of anti-malarial chemotherapy. This is a subject of some importance, since evidence of immunity-related interactions may influence our use of chemotherapy in populations with drug resistance, as well as assessment of the value of suboptimal vaccines. The study aim was to investigate relationship between antibodies and anti-malarial drug treatment outcomes.

**Methods:**

Some 248 children aged 0.5 and 15 years were recruited prior to the high malaria transmission season. Venous blood (5 ml) was obtained from each child to measure antibody levels to selected malaria antigens, using ELISA. Blood smears were also performed to assess drug efficacy and malaria infection prevalence. Children were actively followed up to record clinical malaria cases.

**Results:**

IgG levels to MSP3 were always higher in the successfully treated group than in the group with treatment failure. The same observation was made for GLURP but the reverse observation was noticed for MSP1-19. Cytophilic and non-cytophilic antibodies were significantly associated with protection against all three antigens, except for IgG4 to MSP1-19 and GLURP.

**Conclusion:**

Acquired anti-malarial antibodies may play an important role in the efficacy of anti-malarial drugs in younger children more susceptible to the disease.

## Background

In areas of endemic parasite transmission, protective immunity to *Plasmodium falciparum *malaria is acquired over several years, in response to numerous disease episodes.. *In vitro *studies have shown that antibodies against some malaria vaccine candidates (GLURP, MSP3, and MSP1-19) play a protective role against malaria[1,2]. In addition, epidemiological studies have shown that immunity acquired over several years is strongly related to a drop in mortality and morbidity within populations living in malaria-endemic areas[3]. Although anti-malarial vaccines are being produced and tested, the control of malaria relies heavily on chemotherapy[4,5]. Many of the available anti-malarial drugs are effective, cheap, and easy to distribute. However, in recent years, the increase in drug resistance throughout malaria-endemic regions has been cause for great concern, and has led to calls for the development of new anti-malarial measures, which would involve a larger variety of drug targets as well as a wider array of vaccine strategies[6,7]. In this context, any strategies that maximize the effectiveness of drugs or suboptimal vaccines may lead to significant progress. Among the factors upon which the efficacy of anti-malarial chemotherapy is thought to depend is the patient's immune status[8]. This is a subject of some importance because evidence of interactions may influence our use of chemotherapy in areas with drug resistance, as well as our assessment of the value of suboptimal vaccines. The aim of this study was to investigate whether antibodies can play any direct contributory role in complementing anti-malarial drug therapeutic response, and, if so, whether this was associated with *P. falciparum *malaria treatment outcomes.

## Methods

### Study area and population

Children in this study were aged between 0.5 and 15 years with uncomplicated malaria and were recruited in the village of Balonghin, in the Saponé health district, situated 50 km south of Ouagadougou. The population of Balonghin (approximately 1,600) belongs almost exclusively to the Mossi ethnic group and lives by subsistence farming. The climate is characteristic of the Sudanese savannah, with a dry season from November to May (low transmission season) and a rainy season from June to October (high transmission season). Malaria transmission is markedly seasonal, and most transmission occurs during the rainy season. The main vectors are Anopheles gambiae and Anopheles funestus. *Plasmodium falciparum *is the predominant malaria parasite, accounting for more than 95% of infections in children under five years of age [8]. From February to May, the number of bites per person per night (Entomological Inoculation Rate, EIR) due to *An. gambiae *s.l. is negligible. However, the EIR increases from June to September, then decreases again from September to November and remains low until the next rainy season. The use of insecticide-treated nets in this area is very low, estimated at 1.3%. In addition, the use of indoor residual spraying is non-existent in the area and malaria control mainly relies on treatment of clinical cases[8,9]. To avoid the confounding factor of sickle cell genetic trait, only children homozygous for haemoglobin AA were recruited. The research was given ethical clearance from the National Ethics Committee of Burkina Faso.

### Study design and sample collection

The study design has already been described elsewhere [8]. Briefly, during a cross-sectional survey and prior to the malaria transmission season, each child was seen by a physician. Children exhibiting fever (axillary temperature 37.5°C or higher) were treated presumptively with a standard chloroquine and antipyretic (paracetamol/acetaminophen) drug regimen according to the national drug policy. Five ml of venous blood was withdrawn into an EDTA tube from each target child and the plasma obtained was aliquoted and stored at -20°C for later assessment of antibodies. Thick and thin blood smears were performed by finger-prick for malaria diagnosis. Afterwards, the children were enrolled for a longitudinal follow-up study through biweekly home visits to assess the efficacy of the two drugs, chloroquine and sulphadoxine/pyrimethamine, from early July to October. This study is a sub-cohort of a study which study flow is described elsewhere [8]. Drug allocation was performed randomly using epi-info software. Malaria episode was defined as fever (axillary temperature more or equal to 37.5°C) plus 5,000 *P. falciparum *asexual parasites/μl. The WHO 2003 guidelines were used for assessment of drug efficacy[10].

### Malaria diagnosis

Thick and thin blood films were air-dried, thin blood films were fixed with methanol, and both were stained with 3% Giemsa. Parasite count was performed using microscopy, by experienced technicians. One hundred high power fields were examined and the number of malaria parasites of each species and stage were recorded. The parasite count was expressed as number of asexual parasites per microliter of blood and was calculated assuming a fixed white cell count of 8,000/μl. A slide was declared negative if no parasites were found after 100 high-power field examinations.

### Immunological assessment by ELISA

Antibody assays were performed in a blinded fashion by personnel without knowledge of the microscopic and clinical results of the patients. Levels of specific antibodies (IgG, IgM, and IgG subclasses) were measured by ELISA, using the MSP3 long synthetic peptide (MSP3), recombinant GLURP from *Escherichia coli*, and recombinant MSP1-19 from Baculovirus. The ELISA was done according to the Afro Immuno Assay standard operating procedures (SOP number AIA-007-03; AIA-001-03; AIA-013-03), which have been described elsewhere[11]. In brief, microtiter plates (NUNC - Maxisorp F 96 439454) were coated with long synthetic peptides LR55 MSP3 (1 μg/ml), recombinant GLURP27-500 (0.5 μg/ml) and recombinant MSP1-19 (1 μg/ml), incubated overnight at 4°C, and blocked with 3% dry non-fat milk powder in PBS-Tween 20 for one hour. Plasma samples, diluted 1: 200 (IgG and IgM) or 1: 25 (IgG subclasses), were added in duplicate and incubated at room temperature for two hours. Plates were washed four times between steps. Plates were developed with either peroxidase-conjugated goat anti-human IgG or peroxidase-conjugated goat anti-human IgM (secondary antibody) (Caltag - H10007, H 15007). For IgG subclasses, the secondary antibody was a mouse anti-human monoclonal IgG subclass (Sigma I-9513, clone HP-6002 for IgG1 and IgG2; Sky Bio, M08011, clone ZG4 for IgG3 and Sky Bio, M11014, clone RJ4 for IgG4), and results were visualized using peroxidase-conjugated goat anti-mouse IgG (Caltag M3007). Bound secondary antibody for IgG and IgM and tertiary antibody for IgG subclasses were quantified by colour, using ready-to-use TMB (3, 3', 5, 5'-Tetramethylbenzidine) substrate. Optical density (OD) was read at 450 nm with a reference at 620 nm in a plate reader (Multiskan Ascent, Finland), and the OD values of the test sample were converted into arbitrary units (AU) by means of a standard curve on each plate. The positive controls were from malaria-positive Liberian plasma samples and the negative controls were from Danish plasma samples from Statens Serum Institute (Copenhagen, Denmark).

### Data analysis

Data entry of case report forms for surveillance of malaria episodes was performed using EPI info version 6.0. Data generated from assays in the form of ELISA OD values were converted into Microsoft Excel worksheets using the Auditable Data Analysis and Management System for ELISA (ADAMSEL). Datasets were then transferred to Stata version 8.0 for analysis [12]. Continuous variables were log transformed. Age was coded into categories: 0.5-3, 4-5, 6-10, and > 10 years. Levels of antibodies were compared between age and drug efficacy groups using a Student's *t*-test. Linear regression was fitted to account for the effect of age and sex on the efficacy of the drugs used. Arbitrary units for total IgG and subclasses were log transformed and their geometric means were compared between age categories using ANOVA.

## Results

### Characteristics of the study cohort

The study population consisted of 248 children, aged 0.5 to 15 years, living in the village of Balonghin. The children were selected on the basis of a general census of the village population. They all had AA genotypes for haemoglobin. Regarding gender, 55.2% were male and 44.8% were female. The mean age was 4.07 years. The main malaria species was *P. falciparum*, with a geometric mean of 11,777 (9,182.954-15,104.28) parasites/μL.

### Drug efficacy in the treatment of uncomplicated *Plasmodium falciparum *malaria in the different age groups

**Figure 1:**
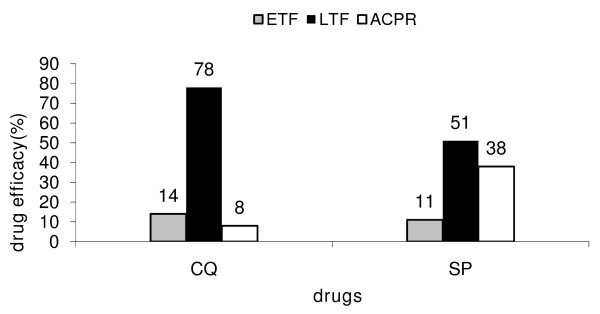
**Chloroquine (CQ) and sulphadoxine/pyrimethamine (SP) efficacy (%)**. ETF: early treatment failure, LTF: late treatment failure, ACPR: Adequate, Clinical and Parasitological Response.

The rate of drug failure (corrected with PCR) in the village of Balonghin (a rural area) was very high for both drugs used during the follow-up period (Figure 1). The rate of early treatment failure (ETF) was 14% for CQ and 11% for SP. The rate of late treatment failure (LTF) was 78% for CQ and 51% for SP. The overall failure rate for both drugs was 92% and 62% for CQ and SP with an Adequate Clinical and Parasitological Response (ACPR) of 8% and 38%, respectively (Figure 1). The failure of both drugs was more pronounced in younger children compared with older ones: 98.3% *vs *47.3% for CQ and 69.2% *vs *0% for SP for younger *vs *older children, respectively.

### Association of antibody levels and efficacy of each drug in the treatment of uncomplicated *Plasmodium falciparum *malaria

To investigate whether the treatment efficacy for each of the drugs assessed was associated with a background of immunity, antibody responses to the investigated antigens (MSP3, MSP1-19, and GLURP) were compared according to each drug's efficacy. Children were assumed to be "protected" if they were able to clear the parasites during the follow-up period, and "unprotected" if they failed to clear the parasites during this period. It appears that in the CQ group, total IgG (3.55 [3.31-3.78] *vs *3.18 [3.12-3.25]; P = 0.01), IgG1 (0.98 [0.53-1.44] *vs *0.44 [0.37-0.52]; P = 0.01), and IgG2 (0.42 [0.23-0.61] *vs *0.21 [0.18-0.25]; P = 0.001) against MSP3 were all strongly associated with drug efficacy. The same trend was observed for IgG (3.91 [3.43-4.39] *vs *0.44 [0.37-0.52]; P = 0.01) and IgM (2.20 [1.41-2.99] *vs *0.97 [0.86-1.08]; P = 0.01) against GLURP. For the SP group, with the exception of IgG3 (1.35 [0.67-2.04] *vs*. 0.67 [0.37-0.97]; P = 0.03) against MSP3, none of the other antigens showed any relationship between efficacy and antibody levels (Table 1). When adjusted for age only IgM to MSP1-19 and GLURP and IgG2 to MSP1-19 in the chloroquine group have shown significant relationship between drug efficacy and antibodies levels.

**Table 1:** Relationship between antibodies levels and drug efficacy in treatment of uncomplicated *P.falciparum *malaria

Antigens	IgG Type	CQ					SP						
	
		Protected GM*(95%CI)	N	Unprotected GM*(95%CI)	N	P	P*	Protected GM*(95%CI)	N	Unprotected GM*(95%CI)	N	P	P*
MSP3	IgG	3.55(3.31-3.78)	16	3.18 (3.12-3.25)	179	**0.001**	0.78	3.42(3.17-3.66)	20	3.26(3.09-3.43)	33	0.27	0.79

	IgM	1.34(1.16-1.53)	16	1.32(1.19-1.45)	179	0.91	0.53	1.48(1.06-1.90)	20	1.40(1.06-1.73)	33	0.75	0.73

	IgG1	0.98(0.53-1.44)	16	0.44(0.37-0.52)	179	**0.001**	0.22	0.58(0.23-0.94)	20	0.56(0.27-0.84)	33	0.89	0.25

	IgG2	0.42(0.23-0.61)	16	0.21(0.18-0.25)	179	**0.001**	0.19	0.29(0.13-0.44)	20	0.18(0.13-0.23)	33	0.09	0.69

	IgG3	1.31(0.64-1.99)	16	0.84(0.67-1.01)	179	0.12	0.48	1.35(0.67-2.04)	20	0.67(0.37-0.97)	33	0.03	0.35

	IgG4	0.04(0.01-0.09)	16	0.01(0.008-0.01)	179	**0.01**	0.29	0.03(0.001-0.08)	20	0.01(0.006-0.02)	33	0.32	0.91

MSP_1-19_	IgG	3.25(2.85-3.65)	16	3.19(3.10-3.28)	179	0.69	0.72	3.11(2.92-3.31)	20	3.22(3.01-3.44)	33	0.47	0.46

	IgM	1.12(0.85-1.39)	16	1.25(1.16-1.34)	179	0.41	**0.01**	1.53(1.20-1.85)	20	1.30(1.09-1.50)	33	0.19	0.77

	IgG1	1.67(0.95-2.38)	16	1.38(1.23-1.53)	179	0.27	0.50	1.38(0.98-1.78)	20	1.20(0.87-1.52)	33	0.47	0.29

	IgG2	0.28(0.16-0.40)	16	0.23(0.19-0.27)	179	0.43	**0.02**	0.22(0.14-0.31)	20	0.19(0.14-0.24)	33	0.48	0.73

	IgG3	0.85(0.54-1.16)	16	0.62(0.54-0.70)	179	0.12	0.85	0.63(0.45-0.81)	20	0.47(0.34-0.60)	33	0.14	0.18

	IgG4	0.03(0.002.0.07)	16	0.03(0.009-0.06)	179	0.95	0.61	0.005(-0.001-0.01)	20	0.04(0.003-0.07)	33	0.14	0.13

GLURP	IgG	3.91(3.43-4.39)	16	3.48(3.38-3.58)	179	**0.01**	0.68	3.58(3.33-3.82)	20	3.47(3.24-3.70)	33	0.54	0.68

	IgM	2.20(1.14-2.99)	16	0.97(0.86-1.08)	179	**0.001**	**0.02**	1.08(0.82-1.33)	20	0.87(0.68-1.05)	33	0.17	0.89

	IgG1	1.39(0.96-1.81)	16	1.05(0.92-1.17)	179	0.13	0.77	1.10(0.77-1.43)	20	1.20(0.84-1.56)	33	0.69	0.36

	IgG2	0.68(0.48-0.88)	16	0.46(0.38-0.54)	179	0.11	0.76	0.54(0.29-0.79)	20	0.50(0.29-0.72)	33	0.82	0.65

	IgG3	0.77(0.59-0.94)	16	0.64(0.57-0.71)	179	0.32	0.34	0.72(0.50-0.94)	20	0.59(0.48-0.71)	33	0.24	0.63

	IgG4	0.006(-0.003-0.16)	16	0.02(-0.002-0.05)	179	0.57	0.30	0.04(-0.03-0.12)	20	0.02(-0.002-0.05)	33	0.63	0.83

N: number of children, GM*: geometric mean of Ig; P: P value not adjusted for age and sex, P*: P value adjusted for age and sex.

### Relationship between age and level of total IgG, IgG subclasses, and IgM responses to MSP3, MSP1-19, and GLURP

The study population was categorized by age group to see whether there was a correlation between antibody levels and age. The antibody levels were compared between age groups using a Student's *t*-test. The general profile of the geometric mean (95% confidence interval) of the antibody levels was found to increase rapidly with age (Table 2), with the following exceptions: IgM to MSP3; IgG1, IgG4, and IgG to MSP1-19; and IgG4 to GLURP. The antigens GLURP and MSP3 responded well, with more than two-fold and three-fold higher antibody levels in older children (> 10 years) than in younger (0.5-3 years) ones, respectively. Cytophilic IgG antibodies (IgG1 & IgG3) were by far more highly expressed than non-cytophilic IgG antibodies (IgG2 & IgG4) (Table 2).

**Table 2:** Relationship between age and level of total IgG, IgG subclasses, and IgM responses to MSP3, MSP1-19, and GLURP

Antigens	Age groups (in years)	N	GM*IgG(95%CI)	GM*IgG1(95%CI)	GM*IgG2(95%CI)	GM*IgG3(95%CI)	GM*IgG4(95%CI)	GM*IgGM(95%CI)
MSP3	[0.5-3]	79	2.96 (2.88-3.04)	0.18 (0.14-0.23)	0.23 (0.21-0.24)	0.29 (0.22-0.37)	0.05 (0.04-0.06)	1.16 (1.06-1.28)

	] 3-5]	69	3.22 (3.13-3.31)	0.24 (0.19-0.31)	0.22 (0.19-0.26)	0.49 (0.38-0.64)	0.05 (0.04-0.06)	0.99 (0.88-1.18)

	] 5-10]	84	3.35 (3.25-3.45)	0.41 (0.33-0.52)	0.28 (0.25-0.30)	0.71 (0.56-0.92)	0.06 (0.05-0.07)	1.21 (1.06-1.39)

	> 10	21	3.51 (3.30-3.70)	0.69 (0.40-1.21)	0.35 (0.26-0.47)	1.05 (0.63-1.75)	0.14 (0.03-0.54)	1.39 (1.12-1.73)

	P		**0,01**	**0,01**	**0,01**	**0,01**	**0,002**	0,10

MSP_1-19_	] 0.5-3]	79	3.13 (0.99-3.28)	1.19 (0.97-1.45)	0.21 (0.18-0.23)	0.40 (0.33-0.48)	0.02 (0.01-0.03)	0.86 (0.77-0.96)

	] 3-5]	69	3.07 (2.95-3.19)	0.97 (0.80-1.17	0.26 (0.24-0.29)	0.48 (0.40-0.57)	0.03 (0.02-0.05)	1.22 (1.11-1.35)

	] 5-10]	84	3.21 (3.07-3.34)	1.02 (0.86-1.21)	0.30 (0.27-0.34)	0.64 (0.54-0.75)	0,03 (0,03-0,09)	1.41 (1.29-1.54)

	> 10	21	3.09 (2.89-3.31)	1.33 (0.96-1.86)	0.35 (0.27-0.45)	0.52 (0.37-0.74)	0.03 (0.02-0.05)	0.98 (0.77-1.25)

	P		0,072	**0.01**	**0.001**	**0.001**	0.09	**0.01**

GLURP	] 0.5-3]	79	3.13 (3.02-3.25)	0.62 (0.51-0.76)	0.29 (0.26-0.32)	0.44 (0.38-0.50)	0.03 (0.01-0.06)	0.54 (0.45-0.65)

	] 3-5]	69	3.41 (3.28-3.55)	0.77 (0.63-0.94)	0.36 (0.32-0.41)	0.55 (0.48-0.63)	0.01 (0.01-0.02)	0.80 (0.72-0.88)

	] 5-10]	84	3.68 (3.53-3.84)	0.84 (0.69-1.09)	0.51 (0.43-0.61)	0.60 (0.52-0.68)	0.05 (0.02-0.09)	0.95 (0.84-1.09)

	> 10	21	3.89 (3.58-4.22)	1.35 (0.96-1.88)	0.64 (0.51-0.82)	0.88 (0.66-1.17)	0.05 (0.02-0.09)	2.00 (1.47-2.73)

	P		**0.01**	**0.01**	**0.01**	**0.01**	0.056	**0.01**

N: number of children, GM*: geometric mean of Ig

## Discussion

The data show high rates of failure for both drugs. The same trend was observed during a follow-up study of the two mentioned drugs in 2003, in Pissy, a neighbourhood in the capital city Ouagadougou (urban area) with LTF levels of 69.5% for CQ and 14.6% for SP[13]. These data, combined with others from several malaria endemic countries, show the real state of resistance to these drugs, which have been used for a long time as first and second line drug treatments for uncomplicated malaria in many malaria endemic countries throughout the world[14,15]. This raises a major point about the emergence of the need for a change in drug policy to a more effective one, such as combination therapy, which is recommended by the WHO guidelines in many malaria-endemic areas[16]. Resistance to anti-malarial drugs is proving to be a challenging problem for malaria control in most parts of the world. Since the early 1960s, the sensitivity of the parasites to chloroquine, the best and most widely used drug for treating malaria, has been on the decline[17]. In the present study, the higher rates of drug failure for both CQ and SP are far above the levels at which the WHO recommends a change in drug policy. In Burkina Faso in 2005, because of the high rates of resistance to CQ and SP, used as first and second line treatments, respectively, the anti-malarial drug policy was changed to combination therapy, as recommended [13]. These results showed that the level of total IgG and IgM against the three antigens, with the exceptions of IgM to MSP3 and IgG and IgM to MSP1-19, was higher in the group of children whose malaria was cured compared with those whose treatment failed, despite similar anti-malarial treatment regimens. A similar finding had already been observed in other malaria endemic areas, in case-control studies[8,18,19], suggesting a supportive role for humoral antibodies in the therapeutic response to anti-malarial drugs. As previously reported [8,18,19], in most cases antibody responses (IgG, IgM and IgG subclasses) to the tested antigens increased with increasing age, in the current paper when adjusted with age as confounding factor, no significant relationship was found between antibodies levels and drug's efficacy except for IgM to MSP1-19 and GLURP and IgG2 to MSP1-19 in the chloroquine group. These results are consistent with previous studies that have suggested a protective role for IgM antibodies against malaria infection[20,21] and for IgG2 associated with lower risk of infection. Nevertheless, evidence that anti-malarial treatment responses in immune patients were always better than those in non-immune patients was first observed by Yorke and collaborators while investigating malaria therapy for general paralysis in early 1942 [18]. The fact that antibodies play an important role in the development of premunition has been well demonstrated [22-24]. Data obtained in the current study have shown that antibody responses, mainly to MSP3 and GLURP antigens, which are predominantly cytophilic isotypes, are associated with protection. Several immuno-epidemiological studies performed in geographically separated areas of Africa have reached the same conclusion; that high levels of two major malaria vaccine candidate antigens (GLURP and MSP3) stimulate production of specific cytophilic antibodies that are significant predictors of protection against clinical malaria[1,11,25]. This provides epidemiological support for the concept that antibodies against GLURP and MSP3 can actively control parasite multiplication *in vivo *through cooperation with cells bearing Fc II receptors [26]. The proof of a protective effect by these two antigens has lead today to a large-scale implementation of a candidate vaccine (GMZ2), which is comprised of a combination of the two molecules (GLURP and MSP3), in children under five years old in several malaria epidemiological settings in Africa. These two distinct mechanisms of parasite clearance (host immunity and drug pressure) may play complementary roles, and may thereby achieve efficacy when one mechanism alone may have been insufficient. This observation has been confirmed in a case-control study in which partial immunity of the patient was shown to play an important role even in the sensitivity of resistant parasites [24].

## Conclusion

In malaria endemic zones, it is of paramount importance to give treatment as soon as the diagnosis is made. But it is not yet clear, however, how early treatment could modify the development of protection to further infections, and knowledge of how a primary infection with the malaria parasite may influence the immune response to later infections is scarce. Nevertheless, these results suggest that a background humoral immune response could be beneficial to children during anti-malarial chemotherapy. This information needs to be taken into account not only for drug treatment policy, but also for malaria vaccine development.

## Competing interests

The authors declare that they have no competing interests.

## Authors' contributions

AD participated in the design of the study, collected data, coordinated the study, performed statistical analysis and wrote the first draft of the manuscript. ABT, AO, ATK, MT, DD, AST and BSS participated in the design of the study, coordinated and participated in the drafting of the manuscript. IN and IS participated in the lab work and data interpretation. All the authors read and approved the final version.
